# GPs’ views and experiences of prescribing non-steroidal anti-inflammatory drugs: a qualitative study

**DOI:** 10.3399/bjgpopen17X100869

**Published:** 2017-05-31

**Authors:** Janet McDonald, Lynn McBain, Anthony C Dowell, Caroline Morris

**Affiliations:** 1 Assistant Research Fellow, Department of Primary Health Care and General Practice, University of Otago, Wellington, New Zealand; 2 Senior Lecturer, Department of Primary Health Care and General Practice, University of Otago, Wellington, New Zealand; 3 Professor of Primary Health Care and General Practice, Department of Primary Health Care and General Practice, and Head of Department, Obstetrics and Gynaecology, University of Otago, Wellington, New Zealand; 4 Senior Lecturer, Department of Primary Health Care and General Practice, University of Otago, Wellington, New Zealand

**Keywords:** non-steroidal anti-inflammatory drugs (NSAIDs), general practice, prescribing patterns, qualitative research

## Abstract

**Background:**

Non-steroidal anti-inflammatory drugs (NSAIDs) are widely prescribed in primary care despite being a high-risk drug group causing significant adverse events, yet little is known about GPs’ perceptions of NSAID risks and benefits.

**Aim:**

To explore GPs’ experiences with NSAID prescribing and views about the risks and benefits of this group of medicines.

**Design & setting:**

A qualitative, inductive study in general practice.

**Method:**

Individual interviews with 15 GPs using a semi-structured interview guide. Interviews were audiorecorded and transcribed. An inductive, thematic approach was used for analysis. Sampling continued until data saturation was achieved.

**Results:**

Three main themes illustrate GPs’ key concerns with managing NSAID risks. The first theme was perceptions of risks and benefits of NSAIDs: GPs expressed differing attitudes towards prescribing medication generally. GPs were aware of the general risks of NSAIDs but weighed these up against specific risk factors and potential benefits for particular patients. They were most concerned about long-term use, risks for children, older people, and patients with comorbidities. The second theme was assessing and mitigating risks when prescribing NSAIDs: GPs considered gastric, cardiac, and renal risks of patients as well as drug interactions. Mitigation strategies included alternative treatment, choice and dose of NSAID, and use of gastroprotective agents. The final theme was other factors impacting on NSAID risks: particularly patient expectations and over-the-counter (OTC) availability.

**Conclusion:**

NSAID prescribing is a complex balance between pragmatism and potential adverse events. Given the costs of morbidity, hospitalisation, and patient demand there is an urgent need to secure a more detailed evidence base and develop practical pathways to support safer prescribing.

## How this fits in

NSAIDs are commonly prescribed in primary care despite their well-known risks. Little is known about GPs’ views of the risks and benefits of NSAIDs or why they may prescribe them in high-risk situations. This study provides an insight into GPs’ experiences with NSAIDs. The findings will inform the development of a tool to assist GPs when they are considering prescribing these drugs.

## Introduction

NSAIDs can be useful in the management of pain and inflammation.^1^ They are used in a range of clinical conditions across all age groups; for example, to manage pain and fever in children, musculoskeletal conditions, gout flares, and osteoarthritis.^2–6^

Alongside their potential usefulness, oral NSAIDs are well known for being a high-risk drug group, especially with regard to gastric, cardiac, and renal adverse events.^7^ They cause particular problems in the older people and those with comorbidities, and interact widely with other medicines.^7^ In children, the level of cardiac risk from NSAIDs is unknown but children can experience gastrointestinal or renal toxicity, the latter being a particular concern in children who may be febrile and dehydrated when given an NSAID.^6,8^

Despite recognised risks, NSAIDs are extensively prescribed in primary care and continue to be linked to high numbers of adverse drug events both in primary care and requiring hospital admission.^9–12^ For example, a survey of 1000 UK GPs found almost all (97%) had had patients who experienced gastrointestinal symptoms from NSAID use, 38% had had patients hospitalised, and 4% reported a patient dying from adverse events due to NSAIDs.^13^ In addition to prescribed NSAIDs, in many countries, some can be purchased OTC at pharmacies or other retail outlets including supermarkets. This contributes to ease of availability, wide use, and potential risk, although retail purchase of NSAIDs is beyond the control of GPs.

Reducing the risk associated with medicines’ use is an issue of high strategic importance globally.^14–16^ NSAIDs are associated with a high risk of medical error and feature in indicator sets developed to reflect potentially inappropriate prescribing.^17–22^

Prescribing information, educational updates, and safety alerts are all available to GPs,^23–24^ but the information provided in these sources is generally either high level and broad or detailed and lengthy, and therefore of limited practical use to a practitioner faced with an individual patient at the point of prescribing. There is currently little knowledge about GPs’ attitudes to NSAIDs or their perceptions of the risks associated with their use.^25^ Two Australian studies in which GPs were interviewed found that while GPs were concerned about patient safety and said they took a cautious approach to prescribing NSAIDs for patients with osteoarthritis, they remained uncertain about the risks and safety of NSAIDs and needed better information to assist with the dilemmas they faced when considering prescribing.^26^ Awareness of medication risks was weighed against the benefits of the drug for the individual patient.^27^

The most commonly prescribed NSAIDs in New Zealand are diclofenac, ibuprofen, and naproxen,^28^ which are all available as subsidised oral formulations in the national pharmaceutical schedule. There are no subsidised selective cyclo-oxygenase-2 (COX-2) inhibitors available, so the patient will need to pay if one is prescribed. A limited number of NSAIDs are available OTC without a prescription, including topical NSAIDs.

The aim of this study was to explore GPs’ experiences with NSAID prescribing and their views about the risks and benefits of this group of medicines in order to better understand the reality and drivers of GPs’ NSAID prescribing. This information will be used to develop resources to assist GPs when they are considering prescribing an NSAID.

## Method

Fifteen face-to-face, semi-structured, qualitative interviews were undertaken with GPs from one large New Zealand city to explore their views and experiences of prescribing NSAIDs. The metropolitan area covered by the study included diverse socioeconomic areas along with inner city and suburban, but not rural, practices.

An advertisement about the study was emailed to general practices in the study area and GPs were invited to contact one of the researchers if they were interested in taking part. Those who responded were sent a full information sheet and interviews were subsequently arranged. After participating, GPs received a $100 NZD gift voucher of their choice in recognition of the time involved in contributing to the study.

Interviews were conducted between February and July 2015. Most (*n *= 10) took place at the GP’s practice, with the remainder at another location convenient to the GP. All interviews were audiorecorded with consent and guided by a semi-structured interview schedule covering three broad areas: perceptions of the risks and benefits of NSAIDs; decision making around NSAID prescribing and what would help with this; and perceptions of patients’ views about NSAIDs. Additional prompts were added as the study progressed in order to further explore developing ideas. Interview recordings ranged from 19 to 65 minutes in length (average 46 minutes). GPs were also asked to complete a short form with demographic data about themselves and their practice population.

During recruitment, the demographics of participating GPs were monitored by the research team in order to achieve a diverse sample of GPs and views (in terms of sex, years of general practice experience, and practice demography). The initial response to the recruitment invitation resulted in a broad sample but one further focused invitation was needed to successfully recruit GPs new to practice. Interviewing ceased when data saturation had been achieved, as evidenced by no new themes arising in the analysis.

Interviews were transcribed verbatim, with all participants offered the opportunity to review their transcript; seven made minor corrections or added comments of clarification. An inductive, thematic approach was used for analysis.^29,30^ Analysis began once the first transcript was completed and continued concurrently with data collection; an iterative research process common in qualitative research.^31^ Transcripts were initially read closely then coded with the aid of NVivo software (version 10). A second researcher also read and commented on all the transcripts and coding notes. Codes were derived from the interview topics as well as additional ideas which arose in the interviews. Where data illustrated more than one concept, they were coded in two or more ways.

As subsequent interviews were coded, new material was compared with existing data for similarities and differences, including looking for instances which deviated from the majority view. The coded data were grouped into themes with summaries and key quotes. These were then discussed among the whole research team, which included practising GPs, a pharmacist, and a qualitative researcher.

## Results

In total, 15 GPs were interviewed; their characteristics are summarised in Table 1. Most (*n *= 13) were working in suburban practices and two in inner city practices. Practice populations ranged from about 1200 to 30 000 and had varied demography, including low, middle, and high socioeconomic status and diverse ages and ethnicities of patients.

**Table 1.** Summary of participants’ characteristics (*n* = 15)

Sex Female Male	9 6
Ethnicity New Zealand European Other	12 3
Years in general practice <5 5–10 11–20 >20	3 2 4 6
Working hours Part-time Full-time	8 7

The three main themes which arose from the data all centred on prescribing risk: perceptions of the risks and benefits of NSAIDs; assessing and mitigating risks; and other factors impacting on NSAID risks.

### Perceptions of risks and benefits of NSAIDs

All the GPs interviewed spoke of the benefits of NSAIDs for managing pain and inflammation. All were cognisant of the risks of this group of medicines but had varying attitudes towards prescribing NSAIDs and medication in general. For example, one described the whole practice as having a conservative prescribing culture while another described their own more liberal attitude towards prescribing (which was also related to less confidence with non-pharmacological pain management):

'So generally our practice sits quite below [the average] for almost all the prescribing … [although] we have about the same [older patients] as other [practices]. I think we try in part to manage in the first instance with non-pharmacological management for all the conditions first.' (GP13)

'I think just with experience and time I’ll probably get more comfortable with saying, “Perhaps you don’t need anything at all. Give yourself 24 hours, 48 hours and then let us know, call the nurses or come back and see me if you’re not right.” And some of the older doctors perhaps do that a bit more, whereas I always feel like you need to give something. And I feel that I would give something, like ibuprofen, quite liberally for fevers, or general pains, or back strain. All those things that you know will come right anyway, but they are here and paying $50 [the consultation fee] and I want to give them something.' (GP7)

GPs weighed the relative risks and benefits for a particular patient but in general, in young, fit patients, and those without comorbidities, benefits were perceived to outweigh risks while there was more concern about risks for children, older patients, and those with comorbidities.

GPs were also more worried about the risks of long-term usage with mixed awareness about the risks of short-term use, with some considering a short course of NSAIDs even in a higher-risk patient:

'I’ve seen a couple of young, fit, healthy people who have ended up in hospital with gastrointestinal bleeds and things, but my thoughts are that in the young, otherwise-fit, healthy person, a couple of weeks of NSAIDs with food, at the appropriate dose, the risks are probably fairly low. It’s when you start getting them in the older people, the people on ACE [angiotensin converting enzyme] inhibitors and diuretics, the diabetics. They’re the ones that I’m sort of a bit hesitant, and then I sort of start to think, “Well this is a real toss-up here” and I often won’t prescribe them.' (GP3)

'I would be relatively happy [prescribing an NSAID to a patient with type 2 diabetes] if it’s an acute thing and there’s quite a short stint of it, although I’m aware it’s a bigger risk in terms of both side effects with GI [gastrointestinal] and renal, but longer term, I wouldn’t be too happy.' (GP14)

For some GPs, the benefit of pain relief from an NSAID was valued quite highly compared with their risks and some trade-offs may be seen as inevitable for patients with complex multimorbidities. Managing high-risk patients also illustrates some of the dilemmas GPs face in practice:

'I always try paracetamol first [with osteoarthritis, then add an NSAID] … People get an idea of what their day’s going to be like, and they don’t wait for the paracetamol to [take effect], you know, they’ll just take it [NSAID]. Which actually I shouldn’t really be insisting they stick to a protocol and put up with pain —﻿ that’s inhumane. If they find a non-steroidal is what gets them through the day, then a non-steroidal is what they should have.' (GP11)

'… particularly in relation to cardiovascular risk and NSAIDs, the belief [behind clinical guidelines] is that longevity is better than quality… [Consider my patient] with dodgy renal function whom I decide to give [an NSAID] for his gout (’cos his diabetes isn’t well controlled at the moment and I don’t want to give him prednisone) and off goes his kidneys and you blame me for an adverse reaction to an NSAID when actually he’s dying of a chronic condition.' (GP1)

Some GPs described changes over time in their NSAID prescribing due to new information about the risk profiles of particular members or formulations of this drug class or through having patients experience adverse events:

'I used to be a great prescriber of diclofenac, but in recent times I’ve really used ibuprofen as my first line on the basis of the decreased cardiac risk using ibuprofen.' (GP8)

'In terms of the slow release preparations, I did a recent reading of them. Now I can’t actually remember whether it was a kidney problem, I think. So I’ve tended to probably not be quite as enthusiastic about using slow release preparations as in the past I had been, so I’m tending to use more immediate release.' (GP8)

### Assessing and mitigating patient risks when prescribing NSAIDs

When GPs were thinking about prescribing NSAIDs, they took into consideration gastrointestinal, cardiac, and renal risks to the patient, interactions with other medication, and the potential to exacerbate asthma or cause a hypersensitivity reaction. Especially concerning were patients with pre-existing conditions and older people:

'… the comorbidities with people who have got diabetes, hypertension, pre-existing renal problems.' (GP3)

GPs were also worried about the use of NSAIDs for children who are febrile and may be dehydrated, increasing their risk of renal damage; they saw themselves as having a role here in educating parents.

'I must admit I’m not a fan of giving ibuprofen to children for fever or to aches and pains … knowing that there are risks with it, and especially if kids are unwell they’re not eating or drinking like they usually do … Plus of course there’s the push to inform parents that they don’t have to be using paracetamol [or other medication] all the time for fevers, and that we should be letting fevers do their work.' (GP10)

Despite broad knowledge of the gastric, cardiac, and renal risks of NSAIDs, there were a few instances where knowledge was lacking. GPs were also uncertain about the magnitude of risks and their application to a specific patient:

'I have a feeling their safety profile [COX-2 inhibitors] might be a bit better for cardiac and things.' (GP7)

'I’m going to assume that somebody who’s sprained their ankle and they’re going to use [an NSAID] for 3 or 4 days, they’re unlikely to have a significant GI bleed in that time. Whereas someone who’s using it every day for months and months is more likely to, so I’m much less concerned about short term use. Same for kidneys too. I don’t know if this is true, but I assume you can’t poison your kidney in 3 or 4 days, it’s probably a chronic thing.' (GP11)

'… so for me, empowering my NSAID prescribing is probably having a better in-depth knowledge about the percentage risks, and about when there’s a grey area that someone’s had a bit of indigestion, a bit of gastritis in the past, and they’re on a bit of aspirin, can I give them 5 or 6 days [of an NSAID] or as an absolutely contra-indicated, shouldn’t I, and should I give them a cover, PPI [proton pump inhibitor] cover? So for me, those are those kind of areas of aaah, what’s going to make me decide that? Well what’s my risk of increased bleeding when I add into that a non-steroidal, add that to somebody who’s already on an anti-coagulant? Should I just completely leave that alone, or … ?' (GP8)

Interactions between NSAIDs and antihypertensive or anticoagulant medication were commonly mentioned by GPs; a few also commented on additional risks if combined with selective-serotonin reuptake inhibitors. In particular, GPs were aware they should avoid the nephrotoxic ‘triple whammy’ of an NSAID, a diuretic, and either an angiotensin converting enzyme (ACE) inhibitor or an angiotensin II receptor blocker (ARB),^32^ but some would consider using an NSAID with either a diuretic or an ACE inhibitor/ARB. Similarly, while anticoagulants were generally viewed as being contraindicated with an NSAID, some would consider prescribing a few days of NSAID in exceptional circumstances. GPs believed adding an NSAID for patients on low-dose aspirin increased the risk of bleeding but this combination was not always considered to be contraindicated.

'So I always avoid the triple, the ACE inhibitor, diuretic, and NSAID combination, but if they’re just on one of those medicines, and they’re otherwise ok, and their renal function’s all right, then I might use it.' (GP3)

'I have prescribed NSAIDs on someone on warfarin; very rarely, but when … we know they’ve got intractable pain and it has worked [in the past], a very short burst, monitor the INR [international normalised ratio], “You know that this might kill you; are you prepared to take that risk? … Take it with food; let’s see how we go.” But I document that I’ve told them ‘life threatening’ so they’re aware. I don’t like doing it, but at the same time, I do feel sorry for people and as long as we’ve made that decision together, that’s fine.' (GP4)

GPs used various strategies to mitigate the risks of NSAIDs, including avoiding NSAID use by considering other medication or treatment options. If they did prescribe an NSAID, they considered which member of the drug class might be safer in view of the patient’s risk factors. Ibuprofen was the most preferred choice because of its lower gastric and cardiac risks, particularly compared with diclofenac. Some prescribed naproxen, especially as a stronger option for gout but also safer than diclofenac. Diclofenac was perceived by some GPs (and also, they reported, some of their patients) as being a more potent NSAID, but also having a higher risk for gastric and cardiac adverse events. There was limited use of COX-2 inhibitors, both because of their cost to the patient (none are subsidised medicines in New Zealand) and doubt by GPs that they offered much benefit over non-selective NSAIDs.

To reduce gastric risk, some GPs would add a proton pump inhibitor (PPI) or H2 antagonist, but there was uncertainty about their effectiveness in preventing NSAID-associated gastritis or bleeding and some were reluctant to add a drug to fix another drug problem:

'It sort of seems ‘Mickey Mouse’ to me to throw in two drugs, you know, giving one drug and then having to cover a problem with another one. That doesn’t seem very good medicine to me.' (GP12)

The cost of adding another medicine (which incurs a prescription charge for the majority of adults in New Zealand) is also a disincentive for some GPs and patients, particularly in lower socioeconomic areas. Other risk reduction strategies were, *' … as with all drugs* [using them] *sparingly, in the lowest possible dose for the shortest possible time.'* (GP11), limiting the quantity prescribed and limiting repeat prescriptions. For older people, GPs considered dose reduction and encouraged ‘as needed’ rather than continuous use. GPs also advised patients about how to take the medication in order to reduce the likelihood of side effects (with food or milk, avoiding alcohol, remaining well hydrated, stopping if they experienced any problems). They discouraged patients from sharing the medication with others.

 When asked what would help to inform their NSAID prescribing, GPs said information comparing the risks of NSAIDs in different situations which was succinct and easy for prescribers to use. There were mixed views about the usefulness of patient handouts, but some GPs thought that short, simple written information would support and reinforce their conversations with patients about NSAIDs:

'I think in terms of your decision tree-type things … how you deal with those ones on the margins is often the most difficult, problematic. You know, when do I stop giving someone who’s diabetic with renal failure NSAIDs and start giving them prednisone instead, which puts their diabetes worse? There’s no, it’s what information do you have to help me with that judgment, so it’s having that in a place that’s easy to find might be useful.' (GP1)

'I’m a big handout giver. Would I give a hand-out about anti-inflammatories? I think it would end up in the bin before they got to the front desk, and I’m also thinking about saving paper. Don’t think that they would be, most patients would not find them useful, but there would be the occasional patient that you would try and drive home your point that it might be useful for … Probably mostly something for the risky people, to explain what their risk is and why their doctor’s cautious about using anti-inflammatories.' (GP11)

### Other factors impacting on NSAID risks

Three other important factors were identified which impact on the risks of NSAIDs: patient experience and expectations, OTC availability of some NSAIDs and the role of pharmacists.

Patients who have used NSAIDs in the past may have preferences for particular NSAIDs based on their experience and this may influence the GP’s prescribing choice. OTC NSAID availability may also influence prescribing:

'I do have a lot of people that swear by long-acting [diclofenac]. And I don’t actually like [diclofenac] that much because my impression is always that it’s a bit harder on the gut, and that certainly naproxen and [ibuprofen] are possibly a little bit safer but then again if people sort of say, “Well naproxen doesn’t work for my gout but [diclofenac] does”, then I’ll usually give them [diclofenac].' (GP3)

'I think sometimes you get middle-aged men who want their [diclofenac] for their sore knee and they’ve got extensive heart history and they’re not managing their cholesterol or their blood pressure or anything, and I guess that’s one situation where I can a bit uneasy about it, but if I've explained all the risks and everything to them, I’m not going to completely restrict them because they can get it over the counter anyway.' (GP6)

GPs thought the OTC availability of NSAIDs influenced patients’ perception of their risks —﻿ *' *[They think] *if you can get it over the counter, they’re completely safe '* (GP2) —﻿ and also meant patients could circumvent a GP’s decision not to prescribe an NSAID by purchasing them at a pharmacy or another retail outlet. GPs had mixed views about whether NSAIDs should be available without a prescription. On the one hand, they perceived this as enabling people to self-manage their health. OTC formulations were noted to be low-dose and their cost may limit their length of use. GPs also thought patients might not be aware of their risks and might unwittingly increase their risks by taking more than the recommended dose or by using more than one NSAID simultaneously (either through purchasing more than one OTC product or using an OTC NSAID in addition to a prescribed one). Hence GPs believed pharmacists have an important role in assessing and advising patients who want to purchase an NSAID, but they were unsure whether pharmacists always had time to do this or knew all the patient’s relevant medical history.

## Discussion

### Summary

This study identifies the complexity involved in NSAID prescribing and the difficult balance that GPs try to maintain between pragmatic patient requests for pain relief and an awareness of potential adverse events. There is a clear message for clinical practice that more support and evidence for both prescribing decision making and information for patients is required. It was found that GPs had a good general knowledge about the risks of prescribing NSAIDs but were uncertain about the magnitude of risk for a particular patient. GPs expressed differing attitudes towards prescribing medication generally, including NSAIDs. Some were willing on occasion to prescribe a short course even in a high-risk situation for their analgesic benefit. Patient expectations and the OTC availability of some NSAIDs also potentially influence GPs’ prescribing decisions.

### Strengths and limitations

The qualitative methodology of this study enabled exploration of GPs’ experiences with prescribing NSAIDs and has provided an insight into their views and challenges. The participating GPs were willing to discuss their NSAID prescribing; it is possible those who chose not to take part would have added other dimensions to the data, but the sample included GPs with varied experience, and they shared examples of prescribing in which they acknowledged potential prescribing risk. It was not possible to include the experiences of GPs in rural or remote areas; it is possible differential access to secondary care may influence their risk management of NSAIDs.

### Comparison with existing literature

The risks and harms of NSAIDs are well known yet they continue to be widely prescribed, even among high-risk patients,^7^ although not all potentially-risky prescribing is inappropriate.^33^ Inappropriate NSAID prescribing was one indicator of hazardous prescribing in the primary care-based PINCER trial,^19^ which demonstrated a pharmacist-led intervention was effective in reducing such prescribing. More recently, Dreischulte *et al* ^34^ focused specifically on high-risk NSAID prescribing in primary care with an intervention consisting of professional education, an informatics tool to identify patients whose NSAID use should be reviewed and a financial incentive to conduct a review of the patient’s notes. Of patients reviewed, 70% needed to be followed up regarding their NSAID use.^34^ Although that study showed there was clearly a need to communicate and negotiate with patients about whether or not they should continue to take NSAIDs, no support was offered to the GPs for managing such conversations. Furthermore, as the patients’ perspective was not reported, their views about having their NSAID medication changed are not known.

The current study found GPs are aware of the general risks of NSAIDs but many were uncertain about specific risks or their magnitude and how to apply their knowledge to the complexity of a particular patient interaction. The next phase of this research will develop tools to support both GPs and patients when discussing NSAIDs, trial these in the practice setting and get feedback from both GPs and patients about their acceptability and usefulness.

### Implications for research and practice

Given the costs of morbidity, hospitalisation, and patient demand there is an urgent need to secure a more detailed evidence base, and develop practical pathways which respond to the realities of daily general practice in order to enable safer prescribing. There is still room for education about the general risks of NSAIDs but further research about predicting risks for particular patients would be useful, though it will be challenging practically and ethically to undertake the necessary trials to gain such information. Tools to support both GPs and patients when discussing the risks and benefits of NSAIDs would also be helpful and we need to know more about how patients perceive such conversations. Greater attention could be paid to the wider systems context that influence safe prescribing.^7,35^ Pharmacists also have an important role to play in relation to both prescribed and OTC NSAIDs.
